# On-Beads Digestion in Conjunction with Data-Dependent Mass Spectrometry: A Shortcut to Quantitative and Dynamic Interaction Proteomics

**DOI:** 10.3390/biology3020320

**Published:** 2014-04-16

**Authors:** Benedetta Turriziani, Amaya Garcia-Munoz, Ruth Pilkington, Cinzia Raso, Walter Kolch, Alexander von Kriegsheim

**Affiliations:** 1 Systems Biology Ireland, Conway Institute, University College Dublin, Belfield, Dublin 4, Ireland; E-Mails: benedetta.turriziani@ucdconnect.ie (B.T.); amaya.garciamunoz@ucd.ie (A.G.-M.); ruth.pilkington@ucd.ie (R.P.); cinzia.raso@ucd.ie (C.R.); walter.kolch@ucd.ie (W.K.); 2 Conway Institute of Biomolecular and Biomedical Research, University College Dublin, Belfield, Dublin 4, Ireland; 3 School of Medicine and Medical Science, University College Dublin, Belfield, Dublin 4, Ireland

**Keywords:** interaction proteomics, in solution digest, label-free quantification, proteomic, mass spectrometry, protein identification, systems biology

## Abstract

With the advent of the “-omics” era, biological research has shifted from functionally analyzing single proteins to understanding how entire protein networks connect and adapt to environmental cues. Frequently, pathological processes are initiated by a malfunctioning protein network rather than a single protein. It is therefore crucial to investigate the regulation of proteins in the context of a pathway first and signaling network second. In this study, we demonstrate that a quantitative interaction proteomic approach, combining immunoprecipitation, in-solution digestion and label-free quantification mass spectrometry, provides data of high accuracy and depth. This protocol is applicable, both to tagged, exogenous and untagged, endogenous proteins. Furthermore, it is fast, reliable and, due to a label-free quantitation approach, allows the comparison of multiple conditions. We further show that we are able to generate data in a medium throughput fashion and that we can quantify dynamic interaction changes in signaling pathways in response to mitogenic stimuli, making our approach a suitable method to generate data for system biology approaches.

## 1. Introduction

The application of mass spectrometry to molecular biology has given big impulses to the field of proteomics and systems biology [1]. The ability to monitor numerous entities simultaneously has shifted the focus of researchers from trying to understand the functions of one single protein, to attempting to decipher a network of proteins regulating cellular functions [2]. Since then numerous strategies have been developed to understand how proteins interact with each other generating signaling networks.

Signaling networks are key elements in all major aspects of cellular life, playing a major role in inter- and intracellular communications. They are involved in diverse processes such as cell-cycle progression, cellular metabolism, cell-cell communication and appropriate response to the cellular environment. Their key mechanism involves the transductions of extra-cellular signals across the cell surface to downstream effectors in the cytosol and the nucleus [3]. 

Signals are commonly transduced through post-translational modifications (PTM) [4]. These include PTMs which alter enzymatic activity, protein stability, structure and localization. Common modifications include phosphorylation, ubiquitination, sumoylation, esterification, alkylation and many others. Due to the multitude of signaling cascades, it is extremely important to decipher which proteins are the key players in these networks and precisely how signals are transmitted and integrated by these molecules [5]. Since the functionality of proteins relies on their ability to interact with one another, pathogenic conditions can reflect the deregulation of such functions. By tracking how protein-protein interactions change dynamically across a network, in response to an extracellular stimulus, it is possible to find/verify how signals travel downstream of the receptor and identify crucial nodes which, if mutated or over-expressed, can lead to pathological transformations. 

Although mass spectrometry is inherently non-quantitative, several approaches have been developed which allow relative and absolute quantification of peptides and by extension, of the proteins they have been derived from, in biological samples. Isotopic labeling of samples/controls is so far the best established method commonly used for absolute and relative quantification. As these techniques have been established for the best part of ten years sophisticated software and methods have been developed and applied. Although elegant, all these methods have particular disadvantages. Metabolic labeling, such as SILAC [6], can essentially be only used in cell culture, although whole animal models have been generated at great cost as well [7]. Other labeling techniques require chemical reactions in order to modify the peptides, which increases the likelihood of error due to efficiencies and handling. More recently label free methods and, crucially, analysis software has been developed which can compete with more traditional labeling methods but without some of the drawbacks, such as cost, handling and the increase in the sample complexity. 

There are two main approaches of label free quantification (LFQ). In the spectral counting approach, protein quantification is achieved by comparing the number of MS/MS spectra identified from each individual protein during the measurement of an entire MS dataset [8]. The number of spectra found for a specific peptide, correlates with the number of identified spectra for each protein and can be translated as a measurement of the final concentration of the protein in the sample. The second approach is based on detecting, identifying and recording ions/peptides with a specific ratio m/z at a specific elution time. Subsequently, the ion, the elution profile and the peak integral are correlated across the samples [9]. In essence, the intensity of each ion is a relative measurement of the peptide concentration in the sample. Protein concentration is then calculated as the sum of all peptide intensities normalized by the size or number of observable peptides.

In this work we present a quantitative interaction proteomic workflow which is suitable for mapping signaling pathways in the context of their dynamic interaction network. The workflow involves the development of a fast protocol to screen protein-protein interactions coupled with a label-free quantification method.

## 2. Experimental Section

**Cell culture:** Human embryonic kidney cell line (HEK293T) and breast cancer cell line (MCF7) were from the American Type Culture Collection (ATCC). Cells were grown in Dulbecco’s modified Eagle’s medium (DMEM) supplemented with 10% fetal bovine serum (FBS) and 2 mM L-glutamine, all the cells were incubated at 37 °C in a humidified atmosphere of 5% CO_2_ in air. 

**Transfection:** Different methods of transfection were tested, among those the Lipofectamine^®^ 2000 (Invitrogen) was the one who showed the best efficiency (data not shown). Cells were plated in a 10 cm dish in DMEM (10% serum) and in the absence of antibiotics. Cells were grown to 70% confluency and transfected with plasmid DNA using Lipofectamine as per manufacturer’s protocol. 1 μg of DNA was transfected into each dish in a mix of 1:6 ratio DNA/Lipofectamine in DMEM containing 0% serum. Five hours after incubation, the media was replaced with fresh DMEM (10% serum). Lysis was performed 48 h after transfection in order to maximize the expression of our tagged-proteins. All the samples were prepared as 3 biological replicates.

**On-beads digestion:** We adapted a previously published protocol [10,11]. More specifically: 

***Day 1*. Immunoprecipitation:** Cells were washed on ice with PBS and collected in a lysis buffer containing 150 mM NaCl, 50 mM Tris-HCl pH 7.5, 2 mM EDTA, 1% NP-40, Phosphatase inhibitor (50 mM NaF, 50 mM Beta-glycero phosphate, 1 mM Sodium Orthovanadate). Lysates were incubated 10 min on ice and then centrifuged at 18.000 g for 5 min at 4 °C. Supernatants containing the proteins were moved into clean 1.5 mL Eppendorf tubes and 10 μL of beads were added to each tube. In our experiment we used anti-FlagM2 agarose (Sigma, Gillingham, UK), anti-eGFP agarose (Chromotek, Munich, Germany), V5 (Invitrogen, Carlsbad, CA, USA) or proteinG-agarose (Sigma, Gillingham, UK) with anti-BAX (Santa Cruz Biotechnology, Santa Cruz, CA, USA). The supernatant was then incubated with the beads for 1 h at 4 °C under end-to-end rotation. In the case of endogenous proteins a total of 1 μg of antibody with 10 μL of proteinG beads was added. At the end of the incubation the beads were centrifuged for 30 s at 5.000 g at room temperature, and supernatant was discarded. In order to remove the detergent and unspecific binders, beads were washed three times by adding 500 μL TBS, the beads were then vortexed, centrifuged for 30 s at 5.000 g and the supernatant was removed. 

**Enzymatic digestion and elution:** Beads were trypsinised, in order to digest the baits and the interacting proteins in 60 μL of Buffer 1 (containing 2 M urea, 50 mM Tris-HCl pH 7.5 and 5 μg/mL Trypsin). Digestion was performed for 30 m 27 °C in a thermomixer, shaking at 800 rpm. After the initial digestion, the samples were centrifuged for 30 s at 7000 rpm and the supernatant was removed and collected into fresh, labelled tubes. Beads were washed twice in 25 μL of buffer 2 (containing 2 M urea, 50 mM Tris-HCl pH 7.5 and 1 mM DTT) and the supernatants were pooled. Samples were left on the bench to continue to digest over night at room temperature.

***Day 2.* Stage Tips Purification:** The following morning, 20 μL of Iodoacetamide (5 mg/mL) was added to the samples and then incubated for 30 m in the dark. Samples were treated with 1 μL trifluoroacetic acid (TFA) to stop the digestion and desalted in C18 stagetips. Briefly, the tips were prepared as described [6], placing a small disc of Empore material 3 M in an ordinary pipette tip, preparing a single tip for each sample. Tips were activated with 50 μL of 50% acetonitrile, 0.1% TFA buffer and washed with 50 μL of 0.1% TFA. 100 μL of sample was added to the column and washed twice with 50 μL 0.1% TFA solution. Liquid was passed through the pipette tip manually with the aid of a syringe or with a light centrifugation step. For both of these steps a (light) centrifugation or the help of a syringe can be used to pass the liquid through the tips. Peptides were then eluted using 25 μL of 50% acetonitrile, 0.1% TFA buffer twice. This last step was performed manually with the help of a syringe. Samples were evaporated in a Speedvac concentrator and re-suspended in 12 μL 0.1% TFA buffer and analysed by mass spectrometry.

**Mass spectrometry analysis:** The tryptic peptides were analyzed on a Q-Exactive mass spectrometer connected to an Ultimate Ultra3000 chromatography system (both Thermo Scientific, Germany) incorporating an autosampler. 5 μL of the tryptic peptides, for each sample, was loaded on a homemade column (100 mm length, 75 μm inside diameter [i.d.]) packed with 1.9 μm ReprosilAQ C_18_ (Dr. Maisch, Germany) and separated by an increasing acetonitrile gradient, using a 40-min reverse-phase gradient (from 3%–32% Acetonitrile) at a flow rate of 250 nL/min. The mass spectrometer was operated in positive ion mode with a capillary temperature of 220 °C, with a potential of 2000 V applied to the column. Data were acquired with the mass spectrometer operating in automatic data-dependent switching mode, selecting the 12 most intense ions prior to tandem MS (MS/MS) analysis. Mass spectra were analyzed using the MaxQuant Software package in biological triplicate and technical replicate. Label-free quantitation was performed using MaxQuant. All the samples were analyzed as two technical replicates and biological triplicates.

**Data analysis:** Data was analyzed using the MaxQuant software package. Raw data files were searched against a human database (Uniprot HUMAN), using a mass accuracy of 6 ppm and 0.01 false discovery rate (FDR) at both peptide and protein level. Every single file was considered as separate in the experimental design; the replicates of each condition were grouped for the subsequent statistical analysis. Carbamidomethylation was specified as fixed modification while methionine oxidation and acetylation of protein N-termini were specified as variable. Subsequently missing values were replaced by a constant (1) in order to allow the following statistical analysis. Results were cleaned for reverse and contaminants and a list of significant interactions determined based on average ratio and t-test.

## 3. Results and Discussion

### 3.1. In Solution Digestion Methods to Overcome Limits of In-Gel Protocols

Interaction proteomic protocols typically include the isolation of the complex, the elution of the bait and its interacting proteins. The eluted sample is then separated on either a one or two-dimensional SDS-PAGE, stained and individual spots/bands are excised. The proteins are then digested in-gel and the peptides are analyzed by LC-MS/MS. This is a laborious and time consuming protocol with each fractionated sample dramatically increasing the machine time required for analysis. Sample fractionation has the additional deleterious consequence of complicating LFQ. In addition, the processing, washing and digesting steps increase the overall level of keratin and other contaminations. Furthermore, the numerous steps required for in-gel protocols can lead to the loss of material, especially for the lower abundant proteins. All these factors can profoundly affect the quality of the samples and compromise the effectiveness of the experiments in general. Pre-fractionation was previously required as the sensitivity of mass spectrometry approaches was limited by technical restrains. The dynamic range, speed and sensitivity of the mass analyzer and the resolution of the HPLC separation are all crucial for the depth and speed of analysis. Recent improvements on the hardware side have dramatically increased the speed and sensitivity of both the LC and mass spectrometer, which has lessened the need for fractionation. As a consequence, numerous in-solution strategies have been developed which bypass these issues and that shorten the time as well as a reduction in handling [10,12]. By combining on-beads digests with a fast and accurate mass spectrometer and an uHPLC front-end separation, we were able to reduce time and handling steps of the experiment, without losing sensitivity in terms of proteins identified per single run. In comparison to previously published methods [10,13,14] we use short a 40 min gradient which we found to be proportionate for the complexity of affinity enriched samples if a Q-Exactive mass spectrometer was used as analyzer.

### 3.2. Label-Free Quantification Is Reliable and a Proficient Tool

Some of the most successful quantitative MS methods are based on metabolic labeling [15,16], among those stable-isotope labeling by amino acids (SILAC) is one of the most used strategies [6,17]. Nevertheless SILAC labeling presents several limitations [18]. First of all, in order to be efficiently labeled, cells must be cultured for at least two weeks in SILAC media. Furthermore, the labeling media are not always tolerated by cells. In addition, SILAC media and isotope labeling chemicals are expensive, therefore limiting their appeal. 

**Figure 1:**
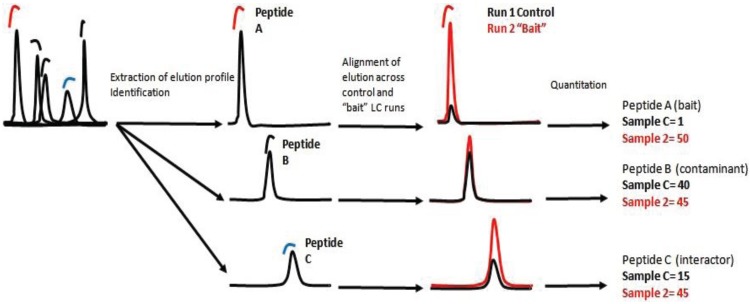
Working principle of label-free quantification (LFQ). Elution profiles of individual ions are mapped; peptide ions are identified by searching fragmentation data (MS/MS) against a database. Protein intensities are calculated by summarizing ion-intensities of peptides uniquely matching the protein sequence identified across all the samples. Individual protein intensities are normalized and the ratio of protein intensities can be calculated. High ratio in “Bait”/Control is an indication of the enrichment in the “Bait” sample, *i.e.*, bait-specific interaction.

Label Free Quantification (LFQ) constitutes a cheap quantification method, suitable for complex biological samples and generation of large-scale datasets [19]. In contrast to metabolic and chemical labeling methods, this technique does not require tailored sample preparation protocols. As such, label-free approaches reduce the time required to set-up experiments, as no specific culture conditions or buffers are required (Figure 1). Compared to SILAC, it is harder to detect small biological fold changes. The samples are analyzed individually and run to run variability must be taken into consideration [20]. On the other hand, LFQ is a sensitive method for the quantification of proteomic data. In contrast to SILAC it does not increase the complexity of the sample and is further low-cost and ideal for the generation of data across multiple perturbations. All these factors render LFQ suitable for generating data used by systems biology. In addition, the developments of new computational tools are constantly improving the reliability and accuracy of LFQ [21].

### 3.3. On-Beads Digestion as a Fast Screen for Protein-Protein Interactions

**Figure 2:**
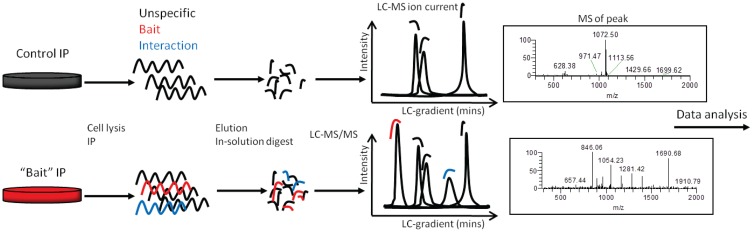
Workflow of the immunoprecipitation (IP) in-solution digestion protocol described. Cells or tissues are lysed; the bait protein is enriched by IP (tagged or endogenous). IPs were washed, eluted, and in-solution digested. Peptides are separated and analysed on a reverse phase LC coupled to a high resolution mass spectrometer.

One of the limitations of established MS experimental protocols is the time required to process the samples. In the classical protocols the samples are washed, eluted, separated by SDS PAGE, stained overnight, excised from the gel, washed, reduced, alkylated and finally digested. This workflow allows the sample to be easily fractionated, thus reducing the complexity of the individual samples prior to the MS analysis. On-bead digests enables the user/researcher to omit all these interim steps and to progress straight to the digestion (Figure 2). The most time consuming step of the proposed on-beads digestion protocol is the incubation of the lysate with the antibody beads, which is about 1 h. After the immunoprecipitation, only three additional handling steps are required prior to the overnight digestion. Firstly, the beads are washed with TBS in order to remove the exceeding/remaining detergent and loosely bound, unspecific, contaminant proteins. This step is crucial for the quality of the samples, since the detergent could interfere with the MS analysis, not only by masking the lower abundant proteins, but also by contaminating the mass spectrometer. Next, the beads are incubated with trypsin in a thermomixer for 30 min and, finally, the peptides are reduced with DTT and collected in a clean tube separate from the beads. The limited digestion is crucial as it avoids the elution of the antibody or proteinG linked from the beads. All these steps require about 1 h, depending on the total number of samples that are processed at the same time. Following the overnight digest, the peptides are alkylated for 30 min, desalted in C18 stage tips, and concentrated in a speed-vacuum centrifuge for about 15–20 min. On the second day, only 2–3 h is required to finalize the process. In total, the hands-on work is reduced to 4–5 h the first day and 2–3 h the second day, the variability is due to the number of samples processed per experiment. Figure 2 shows the experimental scheme. The total mass spectrometry machine-time per sample is reduced to 65 min. This includes sample loading, elution and column regeneration, enabling a throughput of 22 samples per day. The 40 min gradient is adequate and matches the complexity of the sample. The peptides generally elute in less than a 15 s peak off the column. On a Q-Exactive an average 20 K MS/MS are acquired per sample, which roughly translates to 8 K identified individual peptides. 

### 3.4. On-Beads Digestion Protocol Is Suitable for Different Tags and Endogenous Proteins

The protocol proposed is suitable for different experimental conditions. We optimized the method using different affinity Tags and were able to identify the bait and the co-purifying proteins. We conducted preliminary tests in HEK293T with Flag, GFP and V5 tagged proteins. The constructs with different tagged-proteins were transiently transfected and 48 h later the lysates collected. The immunoprecipitation was performed as described. After incubation the samples were washed and prepared for the Western Blot in order to verify the presence of the tagged-proteins (Data not shown). All the tags were expressed and no problems were detected with any of the bait. The following MS analysis confirmed the effectiveness of the protocol for all the different Tag systems. The method is also suitable for screening of protein interactions of endogenous proteins. In this case the results are affected by the specificity and efficiency of the antibody used for the immunoprecipitation, as not all antibodies which may work for western blotting are of good enough quality for this method. We tested some antibodies against low and medium expressed proteins and we were able to corroborate the some of the interactions identified with the exogenous systems (Data not shown).

### 3.5. On-Beads Digestion Is a Sensitive Tool for the Analysis of Interactome

**Figure 3:**
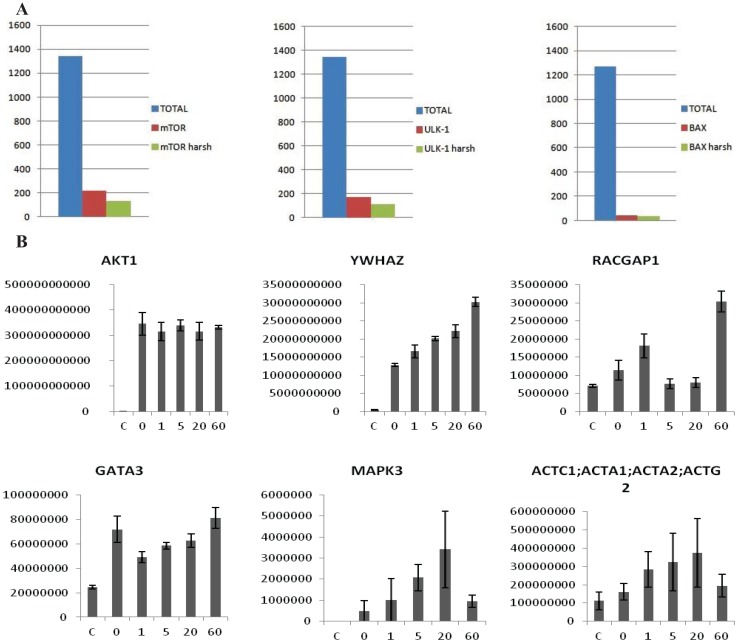
(**A**) Examples of MS analysis using the on-beads digestion protocol. The left panel represents a bar graph of the number of proteins identified in an FLAG-mTor IP from HEK293 cells. The graph shows a comparison of the total amount of proteins identified (blue column), the number of potential interactors after statistical analysis (red column), and the number of potential interactors identified only the IP but not in the control (green column). The panel in the middle side depicts the same analysis for V5-ULK1 as bait. The panel on the right hand side depicts the same analysis for BAX (endogenous) as bait. As shown, an average of 1500 total proteins are identified in both cases, about 50–200 appear to be specific interactors after statistical analysis, and around 40–100 are identified in the IPs alone. (**B**) Dynamic profile of the interaction between Akt1 and select interactors after HRG stimulus in MCF7 cells. MCF7 cells were stimulated with HRG 2 nM for 0, 1, 5, 20 and 60 min. The y-axis represents intensity, the x axis time of HRG-stimulation. The first panel shows that the intensity of the bait (Akt1) is constant across all the points of the time course, while in the other panels show dynamically changing interactors (YWHAZ, RACGAP1, GATA3, MAPK3, ACTC1). Error-bars are SEM, *n* = 6 or 4 (Akt untreated).

The preliminary data in HEK293 showed that the protocol was sensitive and reliable. To verify the broadness and applicability of the protocol we screened the interactome of eleven kinases involved in the ERBB pathway; Akt1, ErbB2, ErbB3, GSK3α, GSK3β, mTor, PDPK1, PRKAA1, S6K1, ULK1 and ULK2. Kinases were tagged with a Flag-tag and transiently transfected into a 10 cm dish of HEK293 cells, the analysis was performed in biological triplicates. Following a 48 h transfection period, the cells were collected and processed as previously described. The eleven Flag-tagged proteins were analyzed singularly/individually and in each run an average of 2000/1500 proteins were identified. This number is comparable to the depth of analysis recently achieved using a data independent SWATH-based mass spectrometric approach [13]. Our method compares favorably with the aforementioned method as we only require a short gradient of 40 min to achieve this depth, rather than 120 min which is required for the SWATH method. From the total interactome a smaller list of potentially specific interactors was isolated after statistical analysis. We used the average ratio and t-test *p*-value in order to identify proteins significantly enriched in the IP *vs*. the control. Proteins showing an average intensity over control ratio higher than 2 and a t-test smaller than 0.05 were considered to be specific. These cut-offs reduced the list of candidates, for all the 10 baits, to 100–150 candidates per bait (Figure 3A). The protocol was also tested in MCF7 cells with the same results (Data not shown). In this case the experiment was performed after treatment with 2 nM Heregulin (HRG) in a five point time course. The aim was to see if our protocol is sensitive enough to observe the dynamical proteomic changes following an external stimulus. Ten Flag-tagged baits were transiently transfected into the cells, 48 h later cells were treated with 2 nM HRG and collected after 0, 1, 5, 20 and 60 min.

**Figure 4:**
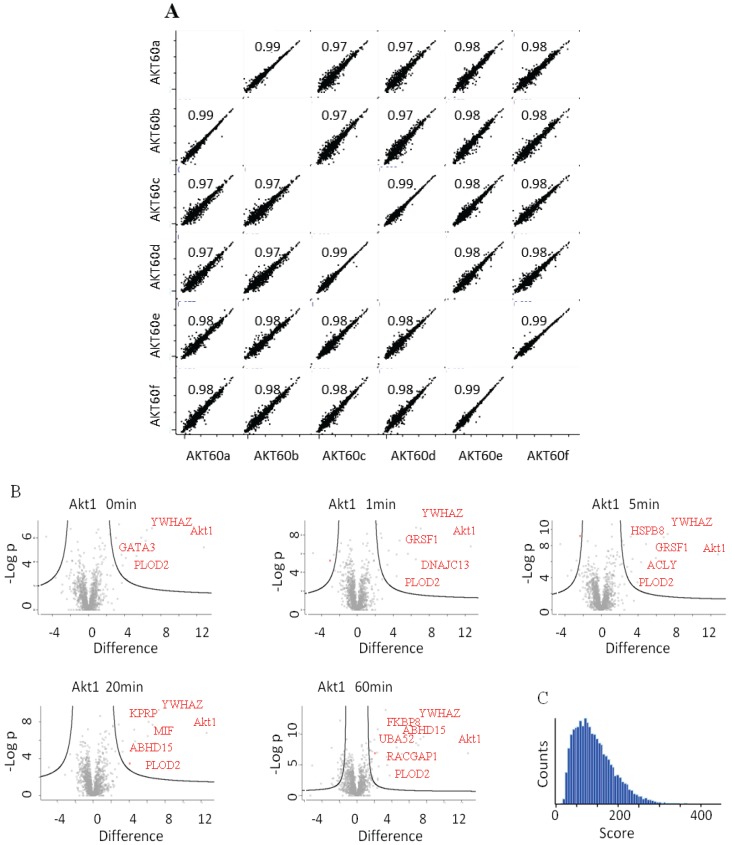
(**A**) LFQ-intensity correlation of the replicates for the Akt1 60' samples, the diagram shows the high correlation not only among the technical but also for the biological replicates. Light blue numbers represent the Pearson’s correlation values for each dot-plot (**B**) Plot of the −Log *p*-value *vs.* the log2 intensity difference of the Akt1 IPs *vs*. the negative control. The potential specific interactors are present in the top right part of the graph. (**C**) Peptides Andromeda-score distribution for the Akt1 time course.

Untransfected cells were used as a negative control and untreated cells as time 0. The experiment was performed in biological triplicate. Each time point was analyzed separately as an independent experiment. After the MaxQuant analysis, LFQ intensities of each time point were grouped and compared to the others. The intensity of some of the interactors was variable along the time points, allowing us to trace a dynamic profile of the interactions (Figure 3B), whereas the intensity of the bait remained constant. According to the data HRG stimulates the interaction between Akt1 and 14-3-3 (YWHAZ) increases steadily, whereas other proteins interact transiently (MAPK3, ACTC1) or dissociate transiently (GATA3). Interestingly, RacGAP1, which was recently identified as Akt substrate [22], appears to interact bi-modally with Akt1 in response to HRG. The quality and reproducibility of the quantification was generally high as illustrated in the LFQ-intensity correlation diagrams. Technical and biological replicates had a Pearson’s correlation coefficient of above 0.99 or 0.97 (Figure 4A). In Figure 4B we plotted the *p*-values *vs*. the log(2) LFQ-intensity ratio of the Akt1/Control IPs. The specific interactions are clustered in the top, right hand section. The left-right hand section consistently showed the presence of GPRC5A and Histones, for which we have no explanation. Figure 4C is a histogram of all the peptide scores deemed to be above the 0.01 FDR.

## 4. Conclusions

Recent technological advances have established mass spectrometry as a reliable tool for studying all aspects of protein biology. This has allowed us to identify and quantify the molecular, protein functions of a cell to great detail and depth. The technical challenge of obtaining accurate data of high coverage limited the applicability of mass spectrometry to a few specialized groups. More recently, democratization has taken place. Thanks to the sturdiness and reliability of modern mass spectrometers, as well as the simplification of the controlling and analysis software, non-specialized groups can now generate data which can compete in terms of depth and accuracy with well-established proteomics facilities. Some stumbling blocks still hamper the broader application of quantitative mass spectrometry across the biological sciences. Specifically, non-expert biologists are taken aback by having to change some of their long established methods for sample preparation. We therefore decided to establish and test a protocol which is as close as possible to traditional, widely used, molecular biological methods. We took our immunoprecipitation protocol, which we have used for years in combination with Western blotting, and added only some wash steps at the end to remove detergents. At this point the samples can be frozen and processed at a later point, allowing for the accumulation of additional samples, such as biological repeats. The samples are then processed without the need of specialized equipment. The simplification of the protocol has allowed us to dramatically extend the base of people considering mass spectrometric approaches within our institute.

A further challenge that prevents biologists to embrace mass spectrometry, as a detection and hypothesis generating tool, is the perceived cost. This stems from an era when, due to pre-fractionation, a single sample required many hours and even days of machine-time. In order to reduce cost and increase throughput we decided to abandon all fractionation steps and to reduce the per-sample machine time to a maximum of one hour, giving us an acceptable throughput, thus reducing cost. Even though we can now run over 20 samples per day, there is clearly a need to further increase this number. If mass spectrometry is to supersede western blotting as the method of choice, it has to compete in terms of sample throughput with SDS-PAGE. New generation, faster mass spectrometers are only part of the answer. Faster and more resolving LC-methods are also required to push this boundary if mass spectrometry is to replace antibody based blotting methods as detection tool.

Although not quite there yet, modern machines have allowed us to perform experiments of such complexity which only a few years ago would have been unfeasible. Our goal is to generate dynamic interaction data from an entire signaling network and to reverse-engineer said network in an unbiased fashion. New mathematical and statistical methods are required in order to achieve this objective but we are confident that the accuracy and simplicity of the described interaction screen will provide us with data of sufficient quality, which is the necessary prerequisite for this endeavor.
